# Cross-Resistance of UV- or Chlorine Dioxide-Resistant Echovirus 11 to Other Disinfectants

**DOI:** 10.3389/fmicb.2017.01928

**Published:** 2017-10-04

**Authors:** Qingxia Zhong, Anna Carratalà, Rachele Ossola, Virginie Bachmann, Tamar Kohn

**Affiliations:** Laboratory of Environmental Chemistry, School of Architecture, Civil and Environmental Engineering, École Polytechnique Fédérale de Lausanne, Lausanne, Switzerland

**Keywords:** environmental virology, virus disinfection, echovirus 11, cross-resistance, water treatment

## Abstract

The emergence of waterborne viruses with resistance to disinfection has been demonstrated in the laboratory and in the environment. Yet, the implications of such resistance for virus control remain obscure. In this study we investigate if viruses with resistance to a given disinfection method exhibit cross-resistance to other disinfectants. Chlorine dioxide (ClO_2_)- or UV-resistant populations of echovirus 11 were exposed to five inactivating treatments (free chlorine, ClO_2_, UV radiation, sunlight, and heat), and the extent of cross-resistance was determined. The ClO_2_-resistant population exhibited cross-resistance to free chlorine, but to none of the other inactivating treatments tested. We furthermore demonstrated that ClO_2_ and free chlorine act by a similar mechanism, in that they mainly inhibit the binding of echovirus 11 to its host cell. As such, viruses with host binding mechanisms that can withstand ClO_2_ treatment were also better able to withstand oxidation by free chlorine. Conversely, the UV-resistant population was not significantly cross-resistant to any other disinfection treatment. Overall, our results indicate that viruses with resistance to multiple disinfectants exist, but that they can be controlled by inactivating methods that operate by a distinctly different mechanism. We therefore suggest to utilize two disinfection barriers that act by different mechanisms in order to control disinfection-resistant viruses.

## Introduction

Waterborne and foodborne viruses are typically efficiently controlled by chemical (e.g., free chlorine and ozone) or physical [e.g., ultraviolet (UV) radiation] disinfectants. However, it is well-documented that viruses may evolve to exhibit tolerance to disinfection. For example, poliovirus isolated from chlorinated drinking water was found to be chlorine-resistant (Shaffer et al., 1980). Similarly, isolates of coxsackievirus B5 from sewage or tap water were more resistant to chlorination compared to their corresponding lab strain (Payment et al., 1985). Finally, resistant viruses can also readily be generated in the laboratory by experimental evolution (Bates et al., 1977; Maillard et al., 1998; Zhong et al., 2016).

While the occurrence of disinfection resistance among virus populations has thus been established, information is lacking regarding the prevalence of such resistant viruses in water distribution system and the environment, or about their overall fitness and contribution to waterborne infections. Given the challenges associated with isolating and identifying such viruses, it is currently unlikely that such information will be routinely obtained in the near future. As such, it appears advisable to design treatment strategies that can control disinfection-resistant viruses, to avoid their proliferation in the first place.

To inactivate a virus, a disinfectant must inhibit one or more of its vital functions, which include host binding, host entry and genome replication. Different disinfectants can target different viral functions. For example, the inactivation of MS2 bacteriophage by UV at 254 nm (UV_254_) is mainly driven by genome damage, which results in the inability of the virus to successfully replicate (Wigginton et al., 2012). In contrast, MS2 inactivation by chlorine dioxide (ClO_2_) is dominated by damage to the protein capsid, leading to the inability of the virus to bind to its host (Wigginton et al., 2012). A possible treatment strategy for viruses with resistance to a given disinfectant may therefore be the application of a disinfectant with a different mode of action (Ballester and Malley, 2004). This approach, however, can only work if a virus does not exhibit cross-resistance to other inactivation mechanisms.

In this work we determined if UV_254_- or ClO_2_-resistant strains of the echovirus 11 (E11) can be controlled by inactivating agents with a different mode of action. Echoviruses are enteric pathogens with clinical manifestations ranging from mild symptoms to more severe diseases such as meningitis, encephalitis, myocarditis, and hemorrhagic conjunctivitis (Knipe and Howley, 2007). They are members of the *Enterovirus* genus, which is frequently detected in the aqueous environment (Fong and Lipp, 2005 and references therein). Due to *Enterovirus'* potential risk to public health via contaminated water (Ford, 1999), this genus was included in the USEPA's Drinking Water Contaminant Candidate List (CCL) (USEPA, 2016). Here, we investigated the susceptibility of resistant E11 populations to treatments commonly applied to control pathogens in water, wastewater and food [free chlorine (FC), ClO_2_, UV_254_, and heat], as well as to an important environmental stressor, namely sunlight.

The action of several disinfectants on members of the *Enterovirus* genus have been previously investigated, yet the dominant inactivation mechanisms remain debated. Different experimental conditions used in different studies with respect to disinfectant concentration, contact time, temperature, pH, or ionic strength, further complicate a comparison of the different findings. For example, Nuanualsuwan and Cliver (2003b) suggested that the poliovirus genome is the primary target of FC inactivation, though in another study conducted at a lower FC working concentration, they observed binding loss for poliovirus and hepatitis A virus (Nuanualsuwan and Cliver, 2003a). Similarly, these researchers reported the genome to be the major target during poliovirus inactivation by UV_254_ (Nuanualsuwan and Cliver, 2003b), though in a different study they also observed binding loss (Nuanualsuwan and Cliver, 2003a). For ClO_2_, the discrepancy in experimental conditions used in different studies also led to a lack of consensus regarding its mode of action. Olivieri et al. (1985) demonstrated that the genomes of inactivated poliovirus were still infectious, thus suggesting that the genome was not the main target of ClO_2_. In contrast, Simonet and Gantzer (2006), who worked with high ClO_2_ exposures (5 mg/L during 120 min), reported that viral RNA did degrade, but did not fully account for inactivation. Genome damage, specifically damage to the 5′ non-coding region, was found to be the main target for the treatment of enterovirus 71 and Hepatitis A virus at ClO_2_ exposures of 13.5 mg/L^*^min or higher (Li et al., 2004; Jin et al., 2013). These authors also reported a similar finding for the inactivation of poliovirus by lower ClO_2_ exposures (0.1–1.2 mg/L during 1–12 min) (Jin et al., 2012). Similarly, an additional study of poliovirus by ClO_2_ at a low ClO_2_ exposure (1 mg/L for 2 min) concluded that a loss in genome replicability was the main mode of ClO_2_ action, whereas loss in host binding was ruled out (Alvarez and O'Brien, 1982). In contrast, our previous work on inactivation of E11 at similarly low ClO_2_ exposures (up to 1.5 mg/L^*^ min) revealed that inactivation coincided with a decrease in host binding, though the loss in this function did not fully account for inactivation (Zhong et al., 2017). Combined, these studies highlight that a comprehensive understanding of the mechanisms of action of these disinfectants, and their dependence on the experimental conditions, is thus still lacking.

Here, we determined the kinetics of inactivation of ClO_2_- or UV_254_-resistant E11 populations by ClO_2_, FC, heat, UV_254_, and sunlight, and compared them to the inactivation kinetics of the corresponding wild-type E11. This allowed us to determine the occurrence and extent of cross-resistance among the disinfection methods tested. We then investigated the main inactivation mechanisms acting on E11 during treatment by these disinfection methods, to evaluate if cross-resistance only affected disinfectants acting by a similar mechanism, or if resistance was a general trait. Finally, we interpreted the results in the context of possible implications for the control of resistant viruses.

## Experimental section

### Cells and viruses

BGMK cells, *Escherichia coli*, E11 and bacteriophage MS2 were cultured and maintained as described previously (Zhong et al., 2016, 2017). Infective E11 concentrations were enumerated as most probable number of cytopathic units per mL (MPNCU/mL), and infective MS2 concentrations were determined as plaque forming units per mL (PFU/mL) (Suess, 1982).

### Resistant echovirus and their corresponding wild-types

**Figure 1:**
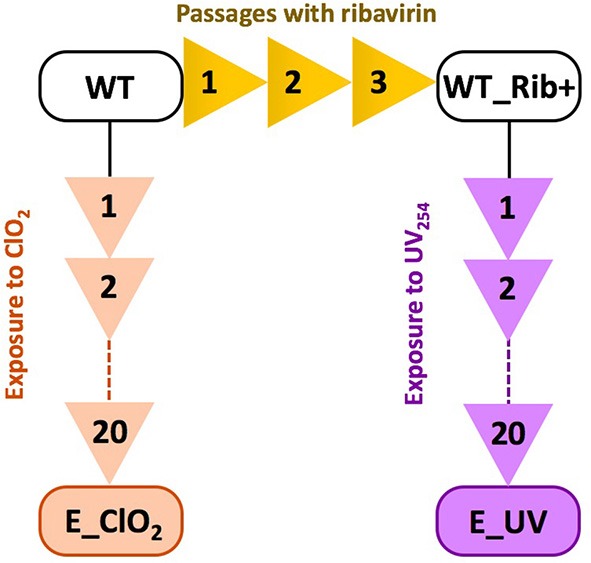
Overview over the experimental evolution lineages to produce resistant populations E_ClO_2_ and E_UV from their wild-types WT or WT Rib+.

ClO_2_-resistant and UV_254_-resistant E11 populations were obtained by experimental evolution (Figure 1). The production of ClO_2_-resistant populations was described in detail in Zhong et al. (2017). Briefly, the E11 laboratory strain, here denoted as “wild-type” (WT), was subjected to 20 passages of directed evolution. During each passage, the virus population was exposed to ClO_2_ up to an exposure of 6 mg/L^*^min, resulting in an inactivation of at least 3 log_10_, before the inactivation was halted and the remaining virus population was regrown on BGMK cells. The resulting ClO_2_-resistant population is henceforth referred to as E_ClO_2_ (“exposed to ClO_2_”). A similar approach was used to obtain UV_254_-resistant E11, except that a different ancestral population of E11 was used. Specifically, prior to any exposure to UV_254_, the E11 lab strain (WT) was first subjected to three cell culture adaptation passages in the presence of the mutagen ribavirin (Fluorochem), to enhance the genetic diversity of the starting population (Crotty et al., 2000). This population (WT_Rib+) was then subjected to 20 passages of titer reduction by UV_254_ followed by regrowth. The details of the UV_254_ setup are given in the following section. The resulting UV_254_-resistant population is named E_UV hereafter. Both evolved populations at their last (20th) passage as well as the corresponding wild-types were sequenced as described below and the mutations of the evolved populations are listed in Table 1.

**Table 1:** Heat map of the frequency of alleles that changed from minor to major or from major to fixed in the evolved populations E_ClO_2_ and E_UV.

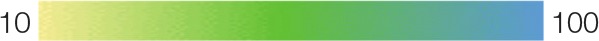				
	**NT**	**AA**	**E_ClO_2_**	**E_UV**
VP4	A849T^a^	Y33F	**0**	**100**
VP2	G1373C^c^	G139R	**100**	**100**
	A2835G^b^	K126R	61	0
	C2844A^a^^,^^d^	P129Q	63	**100**
	T2849A	S131N	60	0
VP1	C2850A	S131N	60	0
	C3162T	T235I	63	0
	A3170G^b^	M238V	63	**100**
	A3233G^b^	K239E	63	**99**
2C	T4200C	V40A	0	**100**
3D	T6006C^d^	M19T	98	**100**
	A6989G	T347A	1	**99**

*Only non-synonymous mutations are included. The location of the mutation, and the resulting change in nucleotide (NT) and amino acid (AA) are listed. Mutations that reached fixation (≥99%) are indicated by bold fonts. Shared major alleles among E_ClO_2_ and E_UV are shaded in gray*.

a These mutations caused an amino acid substitution from ClO_2_-reactive to stable ones;

b These mutations caused an amino acid substitution from FC-reactive to less reactive ones;

c *At this position, the ancestral WT of E_ClO_2_ already has a cytosine as a major allele while its frequency increased by more than 30% in E_ClO_2_. Virus WT_Rib+ has a guanosine*.

d *At these positions, the mutations were already fixed in the ancestral WT_Rib+ of E_UV*.

### Cross-resistance experiments

All inactivation experiments were performed in phosphate-buffered saline [PBS; 5 mM Na_2_HPO_4_ (99%, Acros), 10 mM NaCl (99.5%, Acros), pH 7.4], at a starting E11 concentration of 10^6^–10^7^ MPNCU/mL. The specific inactivation assays associated with the different methods tested are summarized below. Kinetic analyses were performed by monitoring the loss of infective E11 as a function of disinfectant exposure (oxidants), dose (UV_254_ and solar radiation), or time (heat).

#### Chlorine dioxide (ClO_2_)

Concentrated ClO_2_ was produced as described previously (Zhong et al., 2016) and stored in the refrigerator at 4°C. Working solutions were prepared in PBS immediately prior to the experiment.

Inactivation by ClO_2_ was conducted in continuously stirred 10 mL beakers on ice containing 2 mL of PBS and an initial ClO_2_ concentration of 1 mg/L. Samples were withdrawn periodically at time intervals of 10 s to 1 min over the course of up to 5 min, were mixed with sodium thiosulfate (98%, Sigma-Aldrich) to quench the residual ClO_2_, and the virus titer (*N*) was enumerated. Control experiments showed that the addition of sodium thiosulfate did not affect virus infectivity, viral functions, or genome extraction in the subsequent experimental procedures. The ClO_2_ concentration was monitored at the beginning (*C*_*ClO*2, 0_) and periodically throughout the experiment (*C*_*ClO*2_) using the chlorophenol red (Sigma-Aldrich) method described by Fletcher and Hemmings (1985). The ClO_2_ decay throughout the experiment was first order, and the associated decay rate constant *k*_*d*_ (min^−1^) was determined as:

(1) $$\begin{array}{l} \ln\left(\frac{C_{ClO2}}{C_{ClO2,0}}\right)=-k_{d}t \end{array}$$

The ClO_2_ exposure at any time point during the inactivation experiment was estimated from the cumulative area under the curve of *C*_*ClO*2_ vs. *t*:

(2) $$\begin{array}{l} ClO_{2}\;exposure\;=\int_{0}^{t}C_{ClO2}dt \end{array}$$

Kinetic inactivation parameters were obtained by fitting the data to the modified Hom model (Haas and Joffe, 1994):

(3) $$\begin{array}{l} \ln\frac{N}{N_{0}}=-k_{ClO2}C_{ClO2,0}^{n}t^{m}{\left(\frac{1-\exp\left(-\frac{k_{d}t}{m}\right)}{\frac{k_{d}t}{m}}\right)}^{m} \end{array}$$

Here *N*_0_ and *N* are the virus titers at times 0 and *t*, respectively, and *k*_*ClO*2_ is the Hom inactivation rate constant [mg^−n^L^n^min^−m^]. Model parameters *m* and *n* were treated as constant across all experiments (Zhong et al., 2016) and corresponded to 0.30 and 0.46 respectively.

#### Free chlorine (FC)

Inactivation experiments by FC were conducted analogously to ClO_2_ experiments. The initial FC concentrations ranged from 1 to 2 mg/L, and were prepared by diluting NaClO (13–14%, Reactorlab SA) in PBS (pH 7.4). Samples were taken periodically at time intervals of 10 or 15 s over the course of up to 90 s. The FC concentration (*C*_*FC*_) was measured in every sample using the N,N-diethyl-p-phenylenediamine (Sigma-Aldrich) colorimetric method (Rice et al., 2012). The chlorine exposure was determined from the cumulative area under the curve of *C*_*FC*_ vs. *t*:

(4) $$\begin{array}{l} FC\;exposure\;=\int_{0}^{t}C_{FC}dt \end{array}$$

The inactivation rate constant *k*_*FC*_ was then determined using a first-order Chick-Watson model:

(5) $$\begin{array}{l} \ln\left(\frac{N}{N_{0}}\right)=-k_{FC\;}\int_{0}^{t}C_{FC}dt \end{array}$$

#### Ultraviolet radiation (UV_254_)

Continuously stirred 10 mL beakers containing 2 mL PBS (solution depth: 0.6 cm) were spiked with E11 and were placed under a low-pressure 18 W UV-C lamp (TUV T8 Philips) emitting light at 253.7 nm. All solutions were optically dilute, such that the transmission of UV_254_ throughout the reactor was >95%. The fluence rate (*I*_*UV*_) was determined by actinometry using a solution of iodide (Alfa Aesar) and iodate (Acros) in borate buffer (Acros) (Rahn, 1997), and corresponded to 1.7 W/m^2^. The UV_254_ dose was determined from the product of the fluence rate and time (*I*_UV_^*^t). Samples (100 μL) were taken at 1 min intervals over the course of 7 min, and BGMK cells were immediately infected in order to enumerate the concentration of infective E11. The UV_254_ inactivation rate constants (*k*_*UV*_) were determined from model fits of the data to a first-order Chick-Watson model:

(6) $$\begin{array}{l} \ln\left(\frac{N}{N_{0}}\right)=-k_{UV\;}I_{UV}t \end{array}$$

#### Sunlight

Inactivation experiments with sunlight were performed as described elsewhere (Bosshard et al., 2013). In brief, reactors containing 2 mL virus solutions at 5 × 10^4^−−1 × 10^6^ MPNCU/mL were placed under a solar simulator (Sun 2000, ABET Technologies) equipped with a 1,000 W Xenon lamp, an Air mass 1.5 filter, and an atmospheric edge filter. All solutions were optically dilute. The inactivation experiments were conducted in a thermostatic bath at 20°C with magnetic bars constantly stirring the reactors. Samples were taken periodically at time intervals of 1–3 h over the course of 24 h. The solar fluence rate (*I*_*sun*_) was determined by a radiometer (ILT-900-R, International Light) over the range of 290–315 nm and the inactivation rate constant *k*_*sun*_ was obtained by fitting the data to a first-order Chick-Watson model:

(7) $$\begin{array}{l} \ln\left(\frac{N}{N_{0}}\right)=-k_{sun\;}I_{sun}t \end{array}$$

#### Heat

Tolerance to heat was assessed by comparing the decay temperatures of the different E11 populations. Experiments were performed by thermal shift using a PCR thermal cycler (PCR System 9700, GeneAmp). PCR tubes (250 μL) each containing 90 μL of PBS were prepared. Ten microliters of virus solution were injected to the first tube, and this tube was immediately cultured to quantify the starting titer of E11. Starting from 38°C, each thermal shift was set to a 2°increase in temperature. At each shift, one tube containing PBS was preheated in the thermal cycler for 2 min before 10 μL virus solution were injected into the tube. The solution was then kept at this temperature for 1 min and thereafter immediately put on ice until enumeration. Segmental linear regression was applied to determine the decay temperature *Td*, at which the infective virus concentration started to decline at a rate that corresponded to the slope of the second segment (*S*):

(8) $$\{\begin{array}{c} \ln\;(\frac{N}{N_{0}})=0,\;if\;T<Td\\ \ln\;(\frac{N}{N_{0}})=-S\left(T-Td\right),\;if\;T\geq Td \end{array}$$

### Identification of virus functions inhibited by disinfectants

In order to identify the main viral functions affected during inactivation, we quantified the effect of the different disinfectants on genome replication and on host binding. To this end, E11 WT was inactivated by several orders of magnitude by ClO_2_, FC, heat, UV_254_, or sunlight. Isothermal conditions were applied for heat inactivation where viruses were incubated at 56°C in a water bath for 5 min. Samples were collected and divided into two aliquots. The first aliquot was diluted and infectious units of the sample were determined by infectivity assay. The other aliquot was subjected to the genome replication or host binding assays described below.

#### Genome replication

The ability of a genome to replicate after inactivation was examined by quantitative reverse transcription-PCR (qRT-PCR). Viral RNA was extracted from initial and inactivated samples as described previously (Pecson et al., 2009). Prior to extraction, ~10^7^ PFU/mL MS2 was added to each sample as an internal reference to correct for differences in the genome extraction efficiency between the initial and inactivated samples. In each viral extract, the copy numbers of four E11 genome segments of approximate 550-base length each (549–1,080, 2,685–3,254, 4,227–4,793, 5,854–6,364, using primer sets 3F/4R, 11F/12R, 17F/18R, and 23F/24R as specified previously by Zhong et al., 2017) were quantified. Combined, these segments covered ~30% of the E11 genome. In addition, the number of MS2 genome copies in each sample was determined using primer set 5′-CCGCTACCTTGCCCTAAAC-3′ and 5′-GACGACAACCATGCCAAAC-3′ as described previously (Pecson et al., 2009). The extraction efficiency in each sample was calculated as:

(9) $$\begin{array}{l} efficiency=\frac{g\text{\_}MS2}{g\text{\_}MS2_{0}} \end{array}$$

where *g_MS2* and *g_MS2*_0_ are the MS2 genome copy number in the inactivated and the initial samples, respectively. The extraction efficiency in each sample was used to correct the corresponding copy numbers of the different E11 segments. Finally, the intact, PCR-replicable proportion of each E11 genome segment *i* after disinfection, $\left(\frac{g_{i}}{g_{i0}}\right)$, was determined, and was used to estimate the loss in PCR replicability of the whole E11 genome, $\left(\frac{G}{G_{0}}\right)$ using the following extrapolation (Wigginton et al., 2012):

(10) $$\begin{array}{l} \text{log }\left(\frac{G}{G_{0}}\right)=\log\left[{\left(\prod \frac{g_{i}}{g_{i0}}\right)}^{\frac{whole\;genome\;length}{total\;length\;of\;all\;PCR\;segments}}\right] \end{array}$$

Further information pertaining to genome extraction and qRT-PCR quality control can be found in the Supplementary Material.

#### Host binding

Initial and inactivated (by ~5 log_10_) samples were subjected to two binding assays as described in detail elsewhere (Zhong et al., 2017). First, flow cytometry was used to quantify the proportion of BGMK cells with bound viruses before and after inactivation according to a method modified from literature (Triantafilou et al., 2001). This method provided a rapid identification of the disinfecting methods that caused a loss in host binding. Briefly, BGMK cells were harvested and fixed in fixing buffer (4% paraformaldehyde, Alfa Aesar). Cells were then incubated in blocking buffer (PBS with 1% bovine serum albumin, Sigma-Aldrich) for 30 min before virus samples were added and incubated for 1 h at room temperature. After incubation, the solution with unbound viruses was discarded and cells were then sequentially incubated with anti-E11 primary antibody (LSBio) and secondary antibody conjugated with FITC (Sigma-Aldrich) on a rotator. Staining was measured using a CyFlow® SL flow cytometer (Partec) and the proportion of cells with viruses bound was analyzed by counting cells with green fluorescence emitted by FITC using FlowMax.

This flow cytometry assay offers a straight-forward method to track host cells with bound viral capsids. However, the method is less suited for direct quantification of bound viruses, and it cannot distinguish between intact virions and empty viral capsids. Therefore, the flow cytometry results were confirmed and refined by directly quantifying the number of viruses bound to cells before and after disinfection by qRT-PCR. In contrast to the flow cytometry assay, this approach targets the viral genome. Briefly, virus samples were inoculated onto BGMK cell monolayers on ice. After 40 min, any unbound viruses were removed by washing with PBS. The cell monolayer was then subjected to three freeze-thaw cycles and subjected to chloroform treatment. Bound viruses (*N*_*b*_) were harvested and quantified by qRT-PCR as described previously (Zhong et al., 2017) and as further detailed in the Supplementary Material. The difference in qRT-PCR signal between untreated and inactivated virus samples results from a reduction in bound virus, plus the decrease in genome integrity of the targeted segment due to exposure to disinfectants (Wigginton et al., 2012). Hence, the observed binding loss was corrected for the genome decay due to disinfectant exposure. Heat-treated viruses served as a control to assess the extent of non-specific binding of inactivated viruses.

### Genome sequencing

The genomes of virus populations of interest were extracted, and sequencing libraries were prepared as described previously (Zhong et al., 2017). The whole genomes were then sequenced by Next Generation Sequencing (NGS) at the Lausanne Genomics Technologies Core Facility. Briefly, 300 bp PCR amplicons were purified and pooled to obtain 100 ng of nucleic acids per sample. Libraries of 100 bp double-stranded cDNA were constructed using the TruSeq Stranded mRNA Library Prep kit (Illumina). The library nucleic acid concentrations were measured by Nanodrop 2000 (Thermal Fisher Scientific Inc.) and the cDNA quality was checked by Fragment AnalyzerTM (Advanced Analytical). An Illumina HiSeq 2500 platform was used to sequence up to 6 independent, barcoded and pooled libraries. Reads of 100 nucleotides were trimmed and cleaned for further bioinformatics analysis. Only the genomic positions for which the number of reads exceeded 100 were included in downstream analysis. Single-end reads were aligned to E11 Gregory strain using HTS station (David et al., 2014) to call the nucleotide/base at each position The sequence of WT was used as reference to identify the fixed mutations in all evolved populations.

### Statistical analyses

To determine if the evolved (E_ClO_2_ and E_UV) populations were more resistant than their corresponding wild-types, their inactivation rate constants were compared first by paired *t*-test analysis. The comparison of ClO_2_ inactivation kinetics was done by Likelihood Ratio (LR) test (Haas et al., 2014), where the test statistics were determined by Chi-squared distribution table. For all tests the threshold *p*-value for statistical significance was 0.05. The goodness-of-fit was evaluated based on the coefficient of determination (*R*^2^), which was determined by GraphPad Prism (Version 6.01, 2012). Unpaired *t*-test or regular one-way ANOVA was applied to compare all other parameters between populations or treatments.

## Results

### Inactivation of E11 WT by different treatments

**Figure 2:**
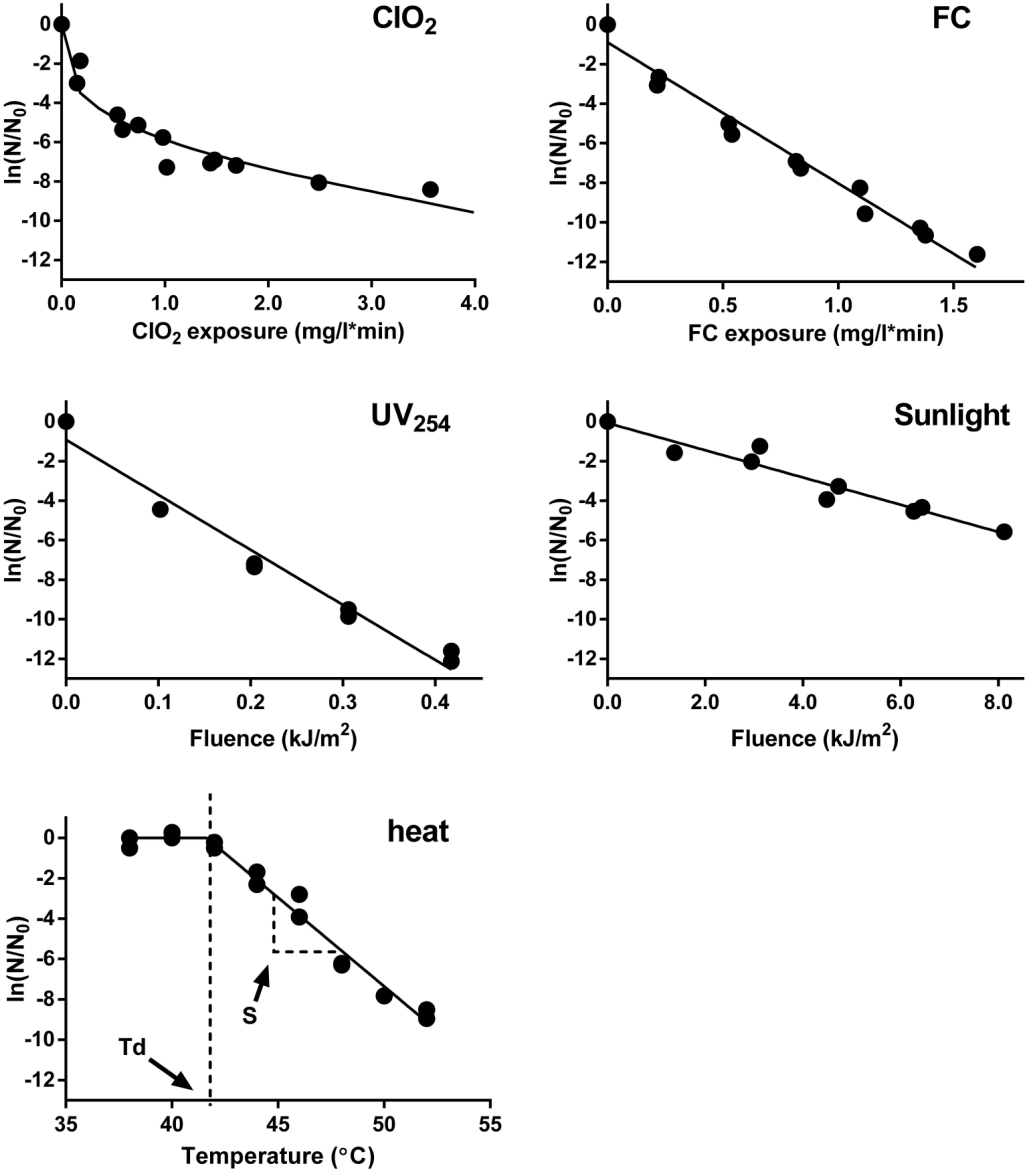
Inactivation of E11 (WT) by ClO_2_, FC, UV_254_, sunlight, and heat. Infectivity loss (ln (*N/N*_0_)) is plotted against disinfectant exposure for ClO_2_ and FC exposure, fluence dose for UV_254_ and sunlight, and temperature for heat inactivation. Experimental data was fitted using the modified Hom model (ClO_2_), Chick-Watson model (FC, UV_254_ and sunlight), and segmental linear regression (heat). Model fits are shown as solid lines. Results from duplicate experiments are presented.

Example inactivation curves of E11 WT by ClO_2_, FC, UV_254_, sunlight, and heat are shown in Figure 2. The inactivation of E11 WT by to FC, UV_254_, and sunlight were first-order with respect to the disinfectant exposure over the inactivation range tested. The inactivation curve of ClO_2_, in contrast, tailed off at higher ClO_2_ exposures. Despite this tail, a 3 log_10_ (99.9%; 6.9 ln) reduction in the infective viral load could be readily achieved (within minutes) by ClO_2_ at the disinfectant exposures tested. Inactivation by FC and UV_254_ at the conditions employed herein were similarly effective. In contrast, inactivation by simulated sunlight proceeded more slowly, such that a 3 log_10_ inactivation required several hours of exposure. Finally, inactivation by thermal shift revealed that the decay temperature of E11 was around 41°C. Beyond this temperature, 3 log_10_ virus decay was achieved within four temperature shifts during which the temperature increased to 50°C.

### Cross-resistance of ClO_2_- and UV_254_-tolerant populations to other disinfectants

**Figure 3:**
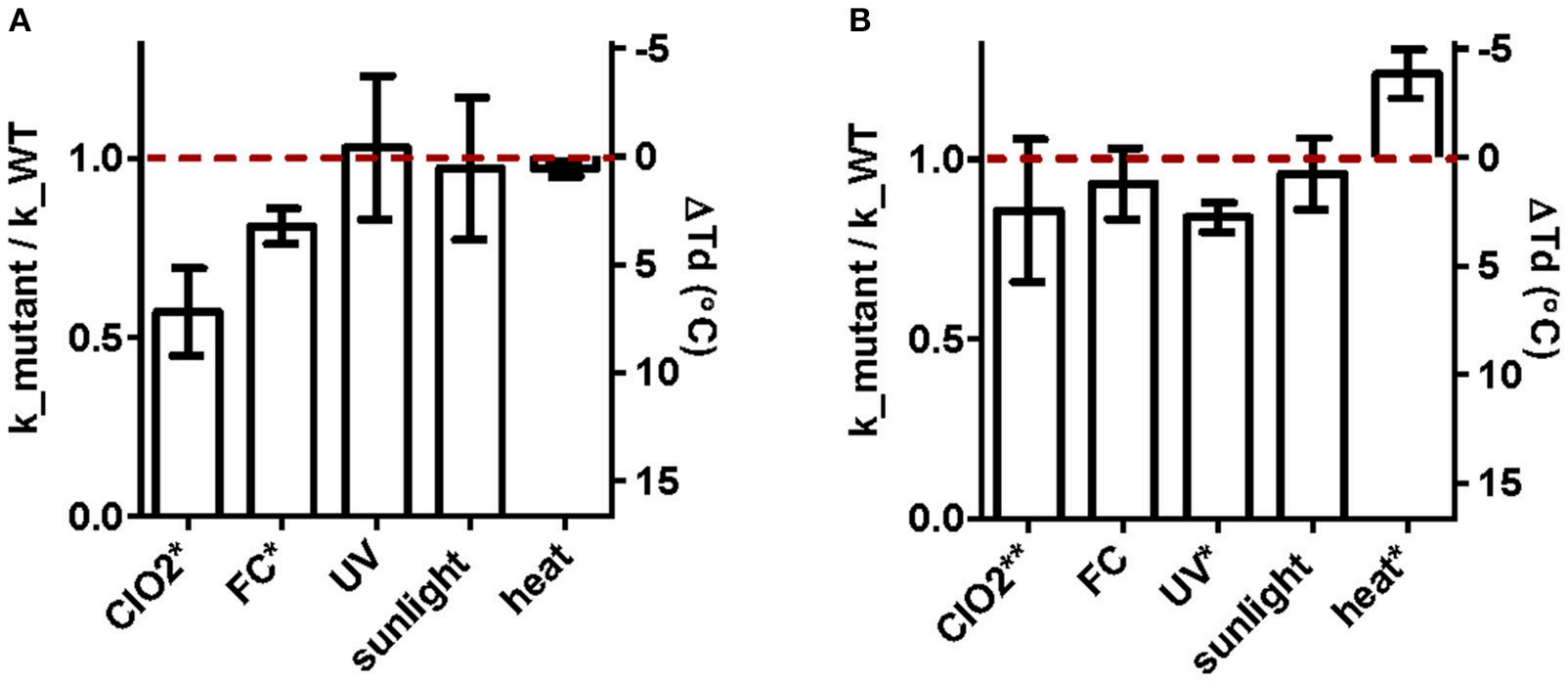
Extent of (cross-) resistance of ClO_2_-resistant populations E_ClO_2_ **(A)** and UV_254_-resistant E_UV **(B)** upon inactivation by ClO_2_, FC, UV_254_, sunlight, and heat. Except heat, results are presented as the ratio of the inactivation rate constants of the resistant populations to those of the corresponding wild-types (left y-axis). Cross-resistance to heat was determined from the differences in the decay temperature (ΔTd) between the resistant populations and their wild-type (right y-axis). The red dashed line indicates unity. Bars extending above the red line correspond to reduced resistance, and bars below indicate enhanced resistance compared to the wild-type. Asterisks indicates significant difference in resistance compared to the wild-type at the 95% (^*^) or 90% (^**^) confidence level. Error bars represent the propagated standard errors from the model fits to pooled duplicate or triplicate experiments.

The susceptibilities of the ClO_2_- and UV_254_-tolerant E11 populations, as well as their corresponding wild-type were tested for the five inactivating treatments considered. Figure 3 shows the extent of resistance to the original stressor (ClO_2_ for E_ClO_2_ and UV_254_ for E_UV), along with the extent of cross-resistance to each additional treatment considered. The data are presented as the inactivation rate constants of the E_ClO_2_ and E_UV populations relative to their respective wild-types, such that a value below unity indicates greater resistance than the wild-type. The absolute inactivation rate constants are reported in Supplementary Tables 1–4. For heat inactivation, the difference in decay temperature to the corresponding wild-type is shown, with the original decay temperatures reported in Supplementary Table 5.

E_ClO_2_ exhibited a 43% reduction in *k*_*ClO*2_compared to its wild-type (*p* < 0.0001; Figure 3A). E_ClO_2_ was furthermore cross-resistant to FC, with a 19% reduction in *k*_*FC*_ compared to its wild-type (*p* < 0.0001). Compared to ClO_2_, resistance to FC was thus less pronounced. Finally, no significant resistance was observed toward UV_254_, sunlight or heat.

E_UV exhibited resistance to UV_254_ inactivation, though its extent was low. Specifically, the reduction in *k*_*UV*_ compared to its wild-type was 15% (*p* = 0.0008; Figure 3B). In addition, E_UV exhibited a 14% reduction in susceptibility to ClO_2_ compared to the wild type, though the difference was not significant at the 95% confidence level (*p* = 0.0977). Finally, this population was more susceptible than its wild-type to heat, yielding a 4°decrease in *Td* (*p* = 0.0070), whereas no measurable resistance to FC or sunlight was observed.

### Viral functions inhibited by different disinfectants

#### Effect of inactivating treatments on genome integrity

**Figure 4:**
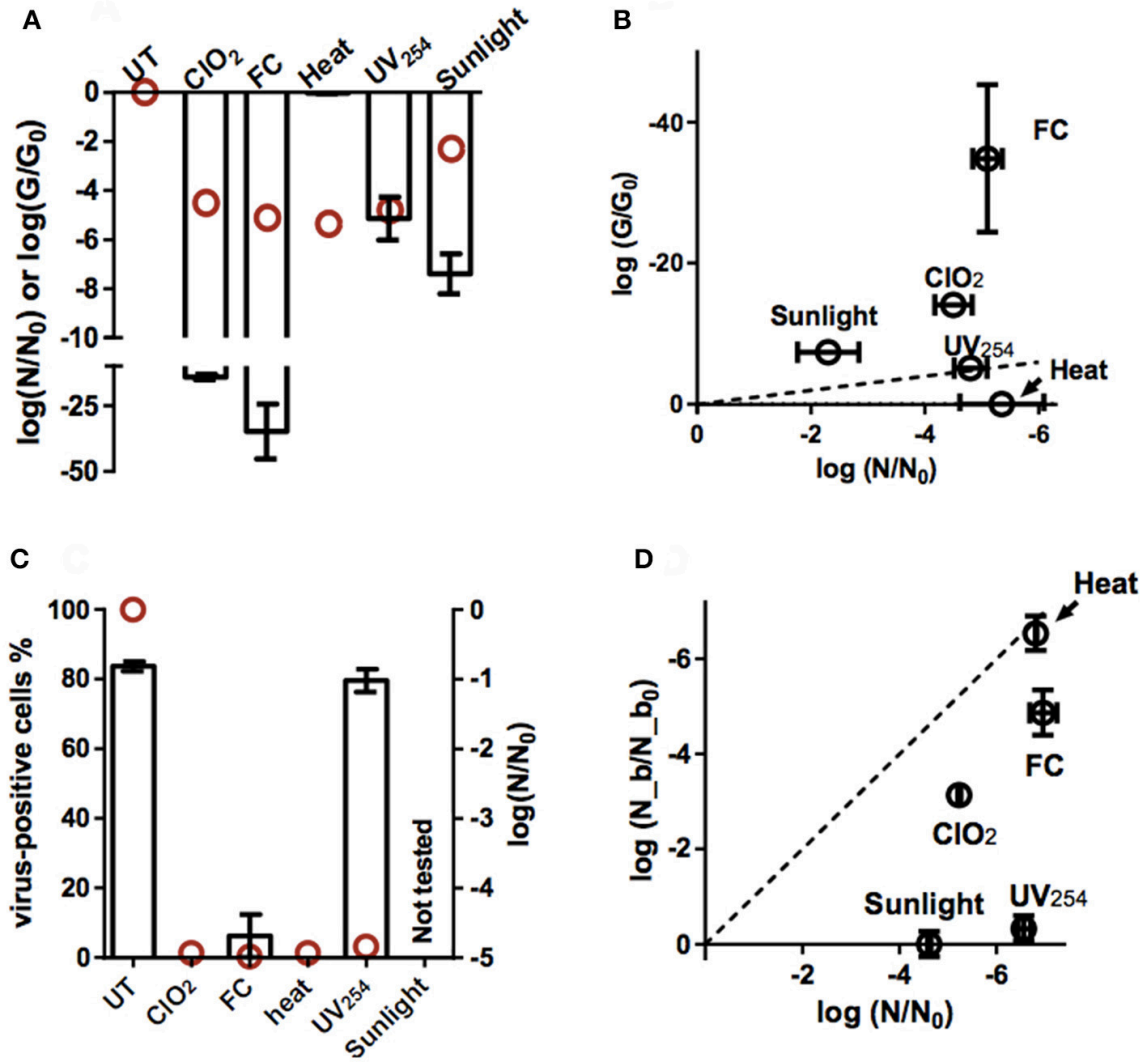
Effect of inactivation by ClO_2_, FC, heat, UV_254_, and sunlight on viral functions. **(A)** Loss of PCR-replicable genome upon inactivation (log(*G/G*_0_); bars). Red circles indicate the corresponding loss in inactivation (log(*N/N*_0_)). Errors bars represent the standard deviations associated with *G/G*_0_ (Ku, 1966). **(B)** Loss of PCR-replicable genome, plotted against infectivity loss. The dashed line represents the 1:1 correlation between genome loss and infectivity loss. Errors bars represent the the MPN enumeration error (horizontal) or standard deviations associated with *G/G*_0_ (vertical). **(C)** Percentage of cells with bound viruses, determined by flow cytometry (bars, left y-axis, UT: untreated E11 sample). Red circles indicate the corresponding virus infectivity loss (right y-axis). **(D)** Residual fraction of bound viruses (log(*N_b/N_b*_0_)) measured by PCR and plotted against infectivity loss. The dashed line represents the 1:1 correlation between binding loss and infectivity loss. Error bars represent the MPN enumeration error (horizontal) or range of duplicate experiments (vertical).

Genome replication by the host cell could not be quantified as an isolated process, as it is preceded by host binding and internalization. We instead determined how the different inactivating treatments affect the ability of the E11 genome to be amplified by PCR. Specifically, the reduction in the qRT-PCR signal upon exposure to ClO_2_, FC, heat, UV_254_, and sunlight was determined for different genome segments (Supplementary Figure 2), and was used to estimate the loss in PCR-replicability of the entire genome (*G/G*_0_, equation 10) of E11 (WT) (Figure 4A).

**Figure 5:**
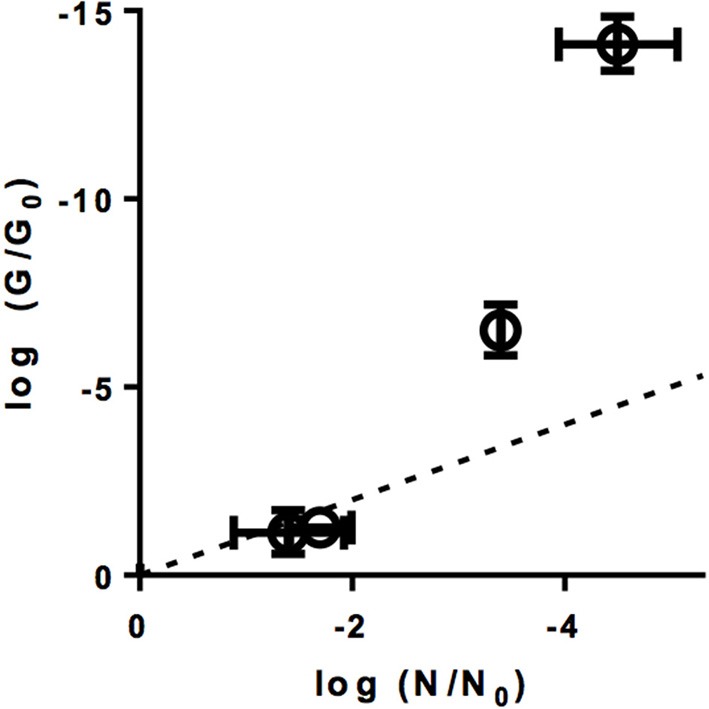
Genome loss log(*G/G*_0_) at different levels of inactivation log(*N/N*_0_) by ClO_2_. The dotted line represents the 1:1 correlation between inactivation and genome loss. Errors bars represent the MPN enumeration error (horizontal) or the standard deviation associated with *G/G*_0_ (vertical) (Ku, 1966).

As is evident from Figure 4A, heat did not cause a measurable loss in PCR-replicable genomes. In contrast, exposure to UV_254_ caused a rapid decrease in *G/G*_0_ and the rate of this decrease corresponded to that of the corresponding decrease in infectivity (Figure 4B). For sunlight-, FC-, ClO_2_- inactivated viruses, the loss in PCR-replicable genome exceeded infectivity loss. Sunlight exposure caused *G/G*_0_ to decrease by 7.4 ± 0.8 log_10_ for a 2.3 ± 0.54 log_10_ of infectivity loss. For FC, the decrease in *G/G*_0_ was 35 ± 11 log_10_, compared to an infectivity loss of roughly 5 log_10_. Finally, for ClO_2_, the loss in genome replicability was further investigated at different levels of inactivation (Figure 5). Interestingly, log *G/G*_0_ roughly corresponded to extent of inactivation (log *N/N*_0_) at low ClO_2_ exposures, but increasingly exceeded inactivation with increasing ClO_2_ exposure. Ultimately, a 5 log_10_ inactivation of E11 infectivity by ClO_2_ resulted in a reduction of log *G/G*_0_ of 14.1 ± 1.0 log_10_.

These data indicate that PCR is more sensitive to genome damage induced by chemical oxidants or sunlight than the host cell. This may be rationalized by considering that the enzymes used in PCR are selected to have a low error tolerance. In BGMK cells, in contrast, only a fraction of the genome damage incurred led to inhibition of genome replication and hence inactivation. Alternatively, genome damage could also be rescued by genome recombination in the cells (Mattle and Kohn, 2012). The extent by which PCR and BGMK cells differ in their ability to replicate damaged genomes disinfectants is currently not well-understood. Despite this limitation of the assay, the PCR results are consistent with a contribution of genome damage, and a resulting loss in the replication function, to inactivation by UV_254_, FC, ClO_2_, and sunlight.

#### Effect of inactivating treatments on host binding

The effect of inactivation on the ability of E11 to bind to BGMK cells was first cursorily screened by flow cytometry. Hereby we determined how inactivation affected the load of viruses bound to BGMK cells. Results revealed that the different disinfection methods affect host binding to varying extents (Figure 4C and Supplementary Figure 1). After roughly 5 log_10_ of infectivity loss by ClO_2_ or heat, no cells carrying viruses could be detected, indicating a strong inhibition of host binding by these two treatments. A small fraction of cells with bound viruses was observed after treatment by FC, though this fraction was below the instrumental limit of quantification. Finally, host binding was only minimally affected by UV_254_ disinfection, resulting in negligible reduction in the observation of cells with bound viruses after treatment.

These findings were further refined by directly quantifying the concentration of viruses bound to cells (Figure 4D) by qRT-PCR. The loss of binding capacity due to heat (6.54 ± 0.36 log_10_) corresponded to the observed infectivity loss (6.81 ± 0.02 log_10_). In contrast, for FC and ClO_2_, the loss in binding was smaller than infectivity loss (4.87 ± 0.48 vs. 7.0 ± 0.28 log_10_ for FC; 3.14 ± 0.10 vs. 5.23 ± 0.03 log_10_ for ClO_2_). Finally, UV_254_ and sunlight manifested less than half a log of binding loss for an infectivity loss was 6.56 ± 0.14 log_10_ and 4.61 ± 0.03 log_10_ respectively.

Assuming a first-order rate of loss in host binding (Wigginton et al., 2012; Zhong et al., 2017), and given the approximate first-order inactivation over the range of disinfection exposures considered, the contribution of binding loss to overall inactivation can be estimated from a log-log plot of binding loss vs. infectivity loss. In Figure 4D, the dotted 1:1 line indicates the region where binding loss can account for all the infectivity loss. The region below the line signifies that binding loss is smaller than infectivity loss, such that other viral functions must also be affected by a given disinfectant. Figure 4D reveals that for heat, binding loss fully accounts for inactivation, whereas for FC and ClO_2_, the contribution to inactivation is ~70 and 60% respectively. Finally, for UV_254_ and sunlight, host binding remains unaffected. Note, however, that the extent of residual binding may be overestimated for most treatments, as it was not possible to control for non-specific binding of inactivated viruses. The only exception is heat, where binding loss was proportional to infectivity loss, such that non-specific binding was unlikely.

### Comparison of the mutation spectrum of the resistant populations

Disinfection resistance arises from mutations to the viral genome which confer an advantage with respect to withstanding disinfection. A comparison of the mutations fixed in each of the resistant populations, along with their likely effects on the viral phenotype, may further aid in understanding the occurrence or absence of cross-resistance.

A total of 12 non-synonymous mutations were identified (Table 1). Of these, five were shared by both resistant populations, and may be a result of adaptation to cell culturing. E_ClO_2_ furthermore exhibited unique non-synonymous mutations in the structural protein VP1, whereas unique non-synonymous mutations in E_UV were located in the replication-related proteins 2C and 3D, as well as in structural protein VP4. Given that there was no cross-resistance of E_ClO_2_ to UV_254_ (Figure 3A) and only minor cross-resistance of E_UV to ClO_2_, these unique mutations serve as candidate mutations responsible for the resistance of E_UV to UV_254_ and of E_ClO_2_ to ClO_2_ respectively.

## Discussion

The occurrence of viral multi-resistance to disinfectants with different modes of action appears limited in the virus populations tested herein. Among the 10 combinations of resistances and disinfectants tested, only one significant cross-resistance was observed between two chemical oxidants, resulting in a reduced sensitivity of ClO_2_-resistant virus to free chlorine. To rationalize the cross-resistance patterns observed, we investigated the mechanisms of action of each disinfectant. Hereby we hypothesized that cross-resistance was a result of a shared mechanism of action of two disinfectants, whereas the absence of cross-resistance is found among treatments acting by different mechanisms. As discussed above, most studies to date report genome damage and inhibition of host binding as the main action of most disinfectants. We therefore focused our mechanistic investigation on these two traits.

### Mechanisms of E11 inactivation

**Figure 6:**
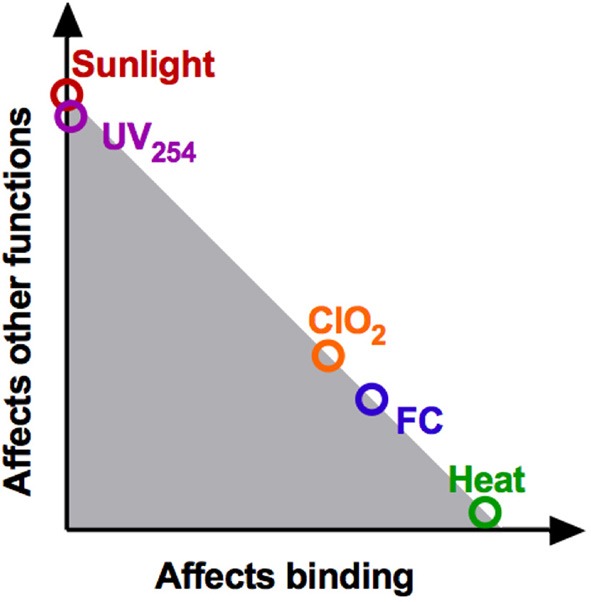
Schematic summary of the contributions of binding loss and other viral functions to overall inactivation. This location of the data points correspond to the ratio of log(*N_b/N_b*_0_): log(*N/N*_0_) (see Figure 4D). The shaded region represents the additive effects of loss of binding, genome replication and potentially other functionalities.

Based on our findings of reduction in genome integrity (Figures 4A,B) and inhibition of host binding (Figures 4C,D), an overview of the main mechanisms of inactivation is presented in Figure 6. Roughly, the modes of action of the different disinfectants can be categorized into three groups, depending on the major viral function impaired. First, for heat, inactivation can be attributed entirely to a loss in host binding. Accordingly, no other viral functions are implicated in inactivation and all genomes remain as replicable as in the untreated samples. Second, for UV_254_ and sunlight, no or minimal binding loss was detected. Inactivation must thus be due to losses in other viral functions, such as genome internalization, replication or virion assembly. While these functions were not tested individually, both UV_254_ and sunlight resulted in a considerable decrease in the fraction of PCR-replicable genome copies. The extensive genome degradation observed by PCR supports the conclusion that the main mechanism of inactivation by both UV_254_ and sunlight involves genome damage, and hence inhibition of replication. Third, inactivation by the oxidants ClO_2_ and FC cannot be attributed to loss in a single virus function. Their mode of action mainly involves a reduction in host binding, yet losses in other functions also contribute significantly. As observed for UV_254_ and sunlight, treatment by FC and ClO_2_ also leads to extensive genome degradation, which likely causes a loss of genome replication.

The proposed mechanisms agree with previous studies on enterovirus that demonstrated that heat and FC inhibited host binding of poliovirus (Nuanualsuwan and Cliver, 2003a) and that UV_254_'s primary target is the genome (Helentjaris and Ehrenfeld, 1977; Nuanualsuwan and Cliver, 2003b). The proposed mechanisms of inactivation of E11 are also are largely consistent with those previously described for MS2 (Wigginton et al., 2012). Major discrepancies were only found for ClO_2_: this disinfectant was previously reported to have no effect on the genome integrity of MS2 (Wigginton et al., 2012), and no effect on host binding for poliovirus (Alvarez and O'Brien, 1982), whereas both these functions were inhibited in E11. The disagreement may be linked to differences in the viral species investigated and their binding motifs, as well as to the disinfectant exposures and solution conditions considered. Furthermore, the inactivation curve of E11 by ClO_2_ exhibits a pronounced tail (Figure 2A). This feature has previously been reported for virus inactivation by ClO_2_, and has been attributed to multiple causes, including the presence of resistant subpopulations or the gradual accumulation of protein oxidation products that form a protective layer on the viral capsid (Berman and Hoff, 1984; Chen and Vaughn, 1990; Thurston-Enriquez et al., 2005; Lim et al., 2010; Jin et al., 2013; Sigstam et al., 2013). The tailing inactivation curve may cause the extent of genome damage by ClO_2_ to not scale linearly with inactivation, but instead to increasingly exceed the extent of inactivation (Figure 5). As such, it is likely that the relative contribution of genome damage to inactivation by ClO_2_ depends on the ClO_2_ exposure and the extent of inactivation considered.

### Cross-resistance of E11 to different disinfectants is specific to the mechanism of inactivation

In the ClO_2_-resistant population E_ClO_2_, we previously identified that resistance was rooted in the ability to utilize an additional host receptor, which was in turn linked to mutations in VP1 (Zhong et al., 2017). This trait allowed the resistant population to better maintain host binding in the presence of ClO_2_, and hence to tolerate higher ClO_2_ exposure. Cross-resistance of E_ClO_2_ may thus be expected to any disinfectant that inhibits host binding (Figure 6). Consistent with this hypothesis, E_ClO_2_ also exhibited resistance to FC. In contrast, no cross-resistance to heat was observed, even though this treatment also affects host binding. This result can be rationalized by considering that ClO_2_ and FC both oxidize viral proteins, whereas heat acts by denaturation (Rombaut et al., 1994; Dodd, 2012). While the mutations in E_ClO_2_ protected the virus from oxidation by allowing alternative receptor use, and by replacing oxidation-reactive by stable amino acids (Table 1) (Sharma and Sohn, 2012), they may not yield the same benefits for protection from denaturation. Finally, the absence of cross-resistance to UV_254_ and sunlight, which do not act on host binding, supports that resistance to ClO_2_ is a mechanism-specific trait.

In population E_UV, resistance to UV_254_ implies a greater ability of the resistant population to deal with mutations accumulated through the action of UV_254_. This ability should also extend to sunlight, since solar UVB (280–315 nm) is also known to have mutagenic action (Pfeifer et al., 2005). Yet, population E_UV did not demonstrate measurable cross-resistance to sunlight. This observation can be explained by considering that the UV_254_-resistance of this population was relatively mild, and that UV at different wavelengths have distinct mutational specificity (Pfeifer et al., 2005).

No cross-resistance of E_UV was observed for heat, which is consistent with its mode of action being entirely protein dependent. Instead, E_UV exhibited enhanced susceptibility to heat. We tentatively attribute this feature to mutation Y33F, which was only found in E_UV, and which is located on the N-terminus of structural protein VP4. The intertwining N-terminus extension of VP1, VP3, and VP4 form a network of protein-protein interactions on the interior of the capsid that is crucial to viral stability (Knipe and Howley, 2007). Therefore, we argue that Y33F on VP4 rendered the E_UV less structurally stable and hence more heat-sensitive. This proposition is supported by the prediction of protein stability changes upon single point mutations using I-Mutant2.0 (Capriotti et al., 2005, 2008). Mutation Y33F was estimated to yield a Gibbs free energy change of −0.76 at 45°C compared to the wild-type. Therefore, Y33F is destabilizing the protein. I-Mutant, however, considers only single proteins, hence free energy calculations that take into consideration inter-protein interactions are needed to validate the result.

Finally, E_UV was slightly cross-resistant to ClO_2_, even though these two disinfectants act by drastically different mechanisms. This finding indicates that populations with a more general resistance spectrum can exist. However, the inverse cross-resistance was not found: E_ClO_2_ remained susceptible to UV_254_. This supports the notion that the multi-resistance of E_UV is not linked to the resistance to UV_254_ *per se*, but may be induced by the experimental evolution assay used to produce the evolved populations. Specifically, resistant populations were produced by repeated and drastic reduction of their population numbers by either ClO_2_ or UV_254_ exposure, followed by regrowth. This action likely selected for those variants that most efficiently proliferated under the experimental conditions used. Efficient proliferation may be aided by enhanced host binding, which in turn is also beneficial to resistance to ClO_2_. Interestingly, both evolved populations shared mutations that are confirmed or probable sites associated with a host receptor switch (VP2-G139R, VP1-M238V, and VP1-K259E; Table 1) (Zhong et al., 2017). This supports the notion that analogous to E_ClO_2_, the ClO_2_ resistance manifested in E_UV is also associated with a better use of an alternative cell receptor.

Given the significant cross-resistance of ClO_2_ and FC observed in E_ClO_2_, the presence of cross-resistance of E_UV to ClO_2_ but absence of cross-resistance to FC is surprising. However, resistance of E_ClO_2_ to FC is less pronounced compared to ClO_2_ (Figure 3A), and cross-resistance to ClO_2_ in E_UV was only slight (Figure 3B). Combined, these two factors likely rendered any cross-resistance of E_UV to FC too small to be experimentally measured.

Overall, this study supports the hypothesis that cross-resistance is mainly found among disinfectants that act by a similar mechanism. To confirm this result, future studies should include viruses with resistance to less specific stressors, such as ozone or free chlorine, which significantly target both viral proteins and genomes. It is conceivable that viruses evolved under pressure of such non-selective disinfectants evolve more general resistances that extend to both genome- and protein-active disinfectants.

### Implications for virus control

As discussed in the introduction, the presence of disinfection-resistant viruses in the environment and is already well-established, though the origin of their resistance is not always known. A potential new source of resistant viruses may be the increasing practice of direct potable reuse of wastewater. In these systems, waterborne viruses may remain in the “treatment-consumption-excretion-treatment” cycle, where they can become subjected to iterate disinfectant exposures and cause new infections. In such a setting, we should be conscious of the potential emergence of disinfection-resistant viruses, and evaluate the best approaches to control their occurrence.

Our results to date suggest that viruses with resistance to a given disinfectant can be controlled by a disinfectant with a different mode of action. This may be achieved by implementing a double disinfection barrier that uses different disinfectants in sequence. From our study on echovirus, a smart choice of disinfectant to include in a double barrier setup is UV_254_. First, UV_254_ is a rather non-selective disinfectant that acts on all genetic material to a roughly similar extent (Lytle and Sagripanti, 2005). This is a stark contrast to a disinfectant like ClO_2_, which only efficiently targets specific amino acids, namely cysteine, tyrosine, tryptophan, histidine, and proline (Tan et al., 1987; Sharma and Sohn, 2012). Compared to ClO_2_, it is thus unlikely that any virus will ever fully escape the pressure of UV_254_. Second, the resistance to UV_254_ was slight compared to that to ClO_2_ (though we cannot exclude that different experimental approaches to produce the resistant virus result in greater resistance). Even if the resistance is minor, however, it remains necessary to include an additional disinfection step using a different disinfectant, such as free chlorine, to control UV_254_-resistant organisms.

The efficiency of different double disinfection barriers to control resistant viruses remains to be tested in future work. In particular, this approach should be validated for additional viruses, as their inactivation mechanisms by the disinfectants tested may differ from that of E11. Furthermore, research should identify ideal combinations of disinfectants and optimal treatment regimes. Ultimately, such a setup should be able to successfully inactivate resistant viruses while avoiding the emergence of multi-resistant viruses.

## Author contributions

QZ and TK designed the experimental plan. QZ, AC, VB, and RO conducted the experiments. QZ, TK, and RO analyzed the data. QZ, AC, and TK wrote the manuscript.

### Conflict of interest statement

The authors declare that the research was conducted in the absence of any commercial or financial relationships that could be construed as a potential conflict of interest.

## Supplementary material

Additional details on the qRT-PCR protocol, figures on analysis of flow cytometer output, reduction of PCR replicable genome segments and absolute inactivation rate constants.
