# Supplementary material for: Cross-Resistance of UV- or Chlorine Dioxide-Resistant Echovirus 11 to Other Disinfectants
Source: Front Microbiol. 2017 Oct 4;8:1928. doi: 10.3389/fmicb.2017.01928 (PMC5632658; doi:10.3389/fmicb.2017.01928)
Supplement: Supplementary file 1 [file DataSheet1.pdf]

Genome extraction and qRT-PCR

All viral samples taken from cell culture were centrifuged at 3500g for 5 min to remove the cell debris before extraction. RNA extraction of 200 µl viral samples was then performed immediately using a PureLink Viral RNA/DNA Extraction Kit (Invitrogen), following the protocol provided by the manufacturer. The kit is specific to viral genomes with a purification step to remove any remaining serum, plasma, cerebrospinal fluid, and cell culture supernatant. Our previous experience demonstrated successful downstream application for the extracted viral genome with this kit and protocol, indicating good nucleic acid quality. The contamination of cellular DNA was excluded during qPCR experiment of samples containing negligible amounts of viral genomes (<0.1%) and with no reverse transcriptase (NRT) controls. The extracted genome samples were stored at -20°C for no more than five days before quantification. We tested the stability and integrity of RNA samples by repeatedly analyzing a sample extracted and stored according to our experimental protocol by qRT-PCR, and confirming that the resulting cycle number did not significantly change. Genome copy numbers were quantified by qRT-PCR in a Mic qPCR Cycler (Bio Molecular Systems) using Primer set of fragment 18 as described previously (Zhong et al., 2017), and a One Step SYBR PrimeScript RT-PCR Kit (Takara). The primers were specific to echovirus 11 Gregory strain and the resulting amplicon size was around 300 bases. The reagent for one reaction was prepared as follows: 7.5µl 2X One-Step SYBR RT-PCR Buffer III, 0.3µl Takara Ex Tap HS (5U/µl), 0.3µl PrimeScript RT Enzyme Mix II, 0.3µl forward primer, 0.3µl reverse primer, 3.3µl RNase Free dH2O and 3µl RNA sample. The following thermocycling conditions were applied: 20 min at 42°C, 15 s at 95°C, 40 cycles of 95°C for 15 s, 55°C for 30 s, and 72°C for 20 s. This was followed by a melting ramp from 72 to 95°C with an increment of 0.2°C/s . No-template controls (NTC) were added to each batch of measurement and were not detectable until 40 cycles. Since we were working with a clean buffer systems, we assume no inhibition in the qRT-PCR step. The purity of nucleic acid was verified by A_{260}/A_{280} ratio using Nanodrop 2000 (Thermal Fisher Scientific Inc.) where the ratios were close to 2.0. The qPCR data analysis was performed using micPCR software version 2.4.0. (Bio Molecular Systems). The efficiencies of amplification were calculated for every experiment (0.895±0.139, median and standard deviation). The r^2 of calibration curve was 0.994±0.012 (median and standard deviation). The specificity of the amplification was monitored in every experiment by melting curve. The limit of detection was estimated according to the method described by Forootan et al. (2017), and corresponded to 2.0 genome copies.

References

Forootan, A., Sjöback, R., Björkman, J., Sjögreen, B., Linz, L., and Kubista, M. (2017). Methods to determine limit of detection and limit of quantification in quantitative real-time PCR (qPCR). *Biomol. Detect. Quantif.* 12, 1–6. doi:10.1016/j.bdq.2017.04.001.

Zhong, Q., Carratalà, A., Shim, H., Bachmann, V., Jensen, J. D., and Kohn, T. (2017). Resistance of echovirus 11 to ClO2 is associated with enhanced host receptor use, altered entry routes and high fitness. *Environ. Sci. Technol.* 51, 10746–10755. doi: 10.1021/acs.est.7b03288
