# Supplementary material for: Cross-Resistance of UV- or Chlorine Dioxide-Resistant Echovirus 11 to Other Disinfectants
Source: Front Microbiol. 2017 Oct 4;8:1928. doi: 10.3389/fmicb.2017.01928 (PMC5632658; doi:10.3389/fmicb.2017.01928)
Supplement: Supplementary file 2 [file DataSheet2.PDF]

Supplementary Tables

Inactivation rate constants and decay temperatures associated with the inactivation of WT, E_ClO_2, WT_Rib+ and E_UV by the different treatments considered. Data from replicate experiments were pooled. The inactivation rate constants, decay temperatures and corresponding standard error of mean were determined from data fitting.

Supplementary Table 1. ClO_2 inactivation rate constants k_{ClO2} [$mg^{-0.46}L^{0.46}$ $min^{-0.3}$].

	k_{ClO2}
WT	6.3±1.2
E_ClO_2	3.6±0.4
WT_Rib+	5.1±1.0
E_UV	4.4±0.5

Supplementary Table 2. FC inactivation rate constants k_{FC} [10^{-2} $Lmg^{-1}s^{-1}$].

	k_{FC}
WT	12.5±0.7
E_ClO_2	10.1±0.3
WT_Rib+	12.8±0.9
E_UV	11.9±1.0

Supplementary Table 3. UV_{254} inactivation rate constants k_{UV} [$kJ^{-1}m^2$].

	k_{UV}
WT	27.9±2.3
E_ClO_2	28.9±4.9
WT_Rib+	27.4±0.8
E_UV	23.0±0.9

Supplementary Table 4. Sunlight inactivation rate constants k_{sun} [$kJ^{-1}m^2$].

	k_{sun}
WT	6.9±0.6
E_ClO_2	8.8±0.3
WT_Rib+	7.5±0.5
E_UV	7.2±0.5

Supplementary Table 5. Decay temperatures (*Td*, °C) of thermal shift experiments.

	Td
WT	41.8±0.3
E_ClO_2	42.2±0.7
WT_Rib+	42.0±1.0
E_UV	38.5±0.5
