# Supplementary material for: Cross-Resistance of UV- or Chlorine Dioxide-Resistant Echovirus 11 to Other Disinfectants
Source: Front Microbiol. 2017 Oct 4;8:1928. doi: 10.3389/fmicb.2017.01928 (PMC5632658; doi:10.3389/fmicb.2017.01928)
Supplement: Supplementary file 3 [file Image1.PDF]

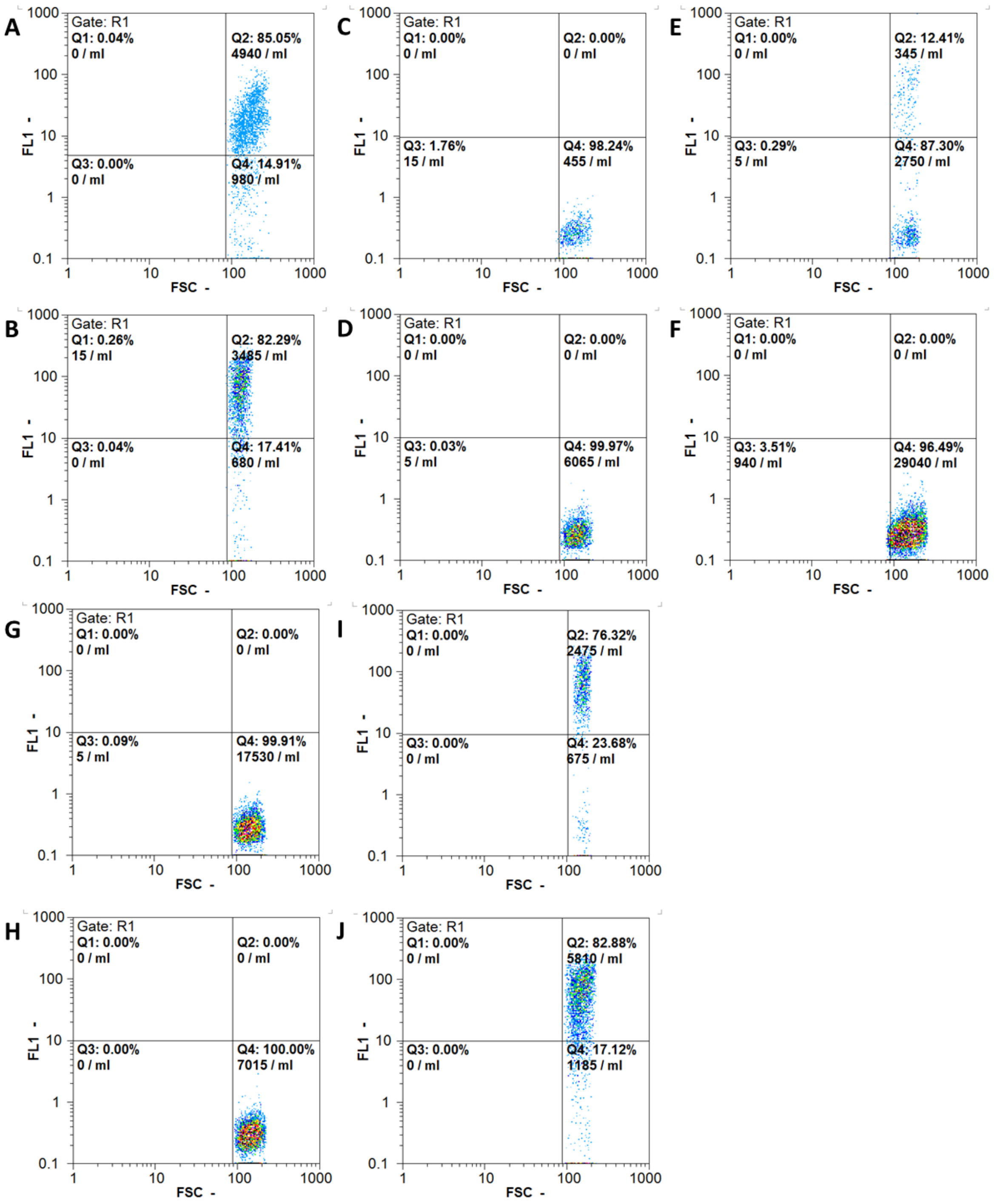

Supplementary Figure 1. Cells with bound viruses before (A, B) and after treatment by ClO_2 (C, D), FC (E, F), heat (G, H) and UV_{254} (I, J). The viruses were stained with FITC, and the results are presented as green fluorescence plotted versus forwards scattering. Cells with bound viruses appear in quadrant Q2. Virus-free cells appear in quadrant Q4.
