# Supplementary material for: Cross-Resistance of UV- or Chlorine Dioxide-Resistant Echovirus 11 to Other Disinfectants
Source: Front Microbiol. 2017 Oct 4;8:1928. doi: 10.3389/fmicb.2017.01928 (PMC5632658; doi:10.3389/fmicb.2017.01928)
Supplement: Supplementary file 4 [file Image2.pdf]

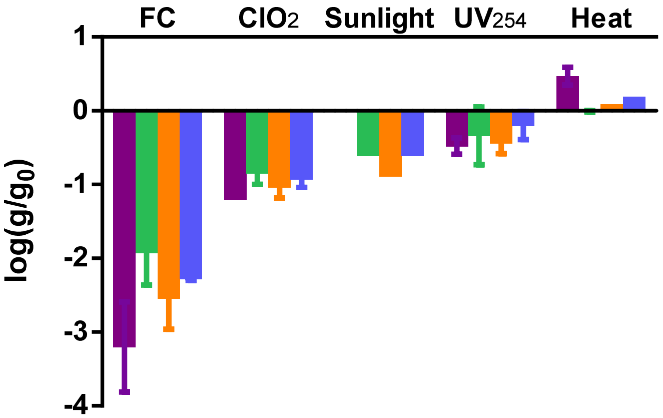

Supplementary Figure 2. Decay of four genome segments (log(g/g_0)) upon inactivation by FC, ClO_2, sunlight, UV_{254} and heat. Segment 1 was not tested for decay by sunlight. Error bars represent the standard deviation of triplicate experiments.
